# Nationwide profiling of vaginal microbiota in Chinese women reveals age‐dependent shifts and predictive biomarkers for reproductive health

**DOI:** 10.1002/imt2.70088

**Published:** 2025-10-23

**Authors:** Cancan Qi, Yingxuan Zhang, Wei Qing, Rongdan Chen, Zuyi Zhou, Yumei Liu, Enzhong Chen, Wenyi Chen, CALM2004 Consortium Investigators, Hongwei Zhou, Muxuan Chen

**Affiliations:** ^1^ Microbiome Medicine Center, Department of Laboratory Medicine ZhuJiang Hospital, Southern Medical University Guangzhou China; ^2^ Guangdong Provincial Clinical Research Center for Laboratory Medicine Guangzhou China; ^3^ Department of Obstetrics and Gynaecology, Shenzhen Hospital Southern Medical University Shenzhen China; ^4^ State Key Laboratory of Organ Failure Research Southern Medical University Guangzhou China; ^5^ Department of Laboratory Medicine, Shenzhen Eye Hospital Shenzhen Eye Medical Center, Southern Medical University Shenzhen China

## Abstract

The vaginal microbiome is central to reproductive health, yet large‐scale studies in East Asian populations remain scarce. Here, we characterized the vaginal microbiota of 6423 Chinese women of reproductive age across 18 provinces and assessed associations with 33 host factors. We observed a striking compositional transition around age 40, marked by declining *Lactobacillus crispatus* and enrichment of dysbiosis‐associated taxa including *Gardnerella vaginalis*, independent of lifestyle or sociodemographic influences. Sexual behavior, contraceptive use, and educational attainment emerged as key determinants of community structure, differentially shaping *Lactobacillus crispatus* and *Lactobacillus* iners. Despite these associations, host factors explained less than 2% of overall variation, highlighting the resilience and individuality of the vaginal microbiome. To quantify vaginal health, we derived a microbiome balance score, validated it in external cohorts, and demonstrated its predictive power for incident bacterial vaginosis and sexually transmitted infections. Our findings establish a national‐scale reference for the vaginal microbiome in Chinese women, reveal a midlife inflection point in microbial composition, and introduce a clinically actionable metric for risk stratification. These insights advance mechanistic understanding of host–microbiome interactions and inform strategies for precision interventions to preserve vaginal health.

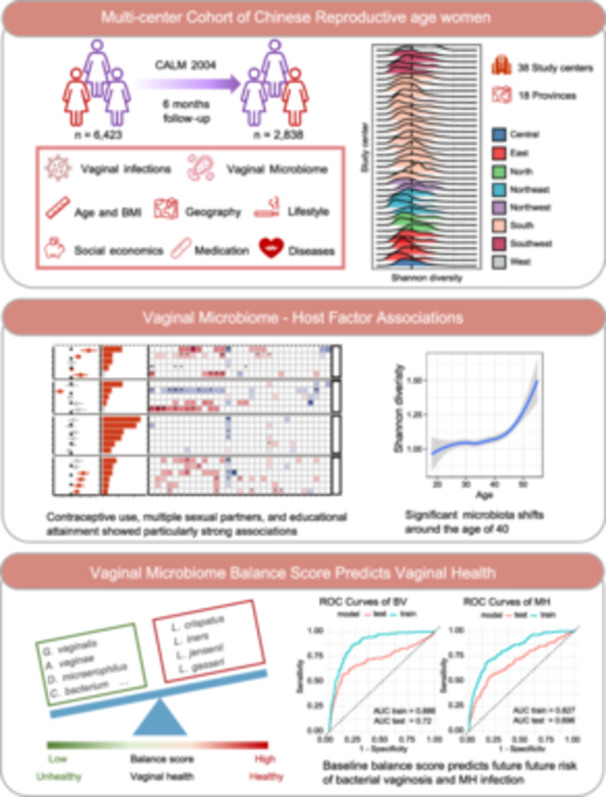

To the Editor,

The vaginal microbiome plays a critical role in women's reproductive health, with dysbiosis linked to adverse outcomes such as sexually transmitted infections (STIs) [1], spontaneous preterm birth [2], and even cervical lesions [3]. Even among healthy women, its composition exhibits substantial variability, influenced by factors including ethnicity, menstrual cycle, and lifestyle [4, 5]. Such variability may contribute to inconsistencies in reported determinants and clinical implications across different populations [6, 7]. However, most studies conducted in East Asian women, including those in China, have been limited in scale and rarely examined a broad spectrum of host factors, thereby restricting comprehensive insights into the determinants of the vaginal microbiota and their relevance to reproductive health.

In this study, we profiled the vaginal microbiota of over 6000 Chinese women of reproductive age and investigated its associations with a wide range of host factors, including demographic and geographical characteristics, lifestyle, reproductive history, medication and disease history, as well as vaginal and cervical clinical symptoms. Our objectives were to quantify the relative contributions of these factors to microbiota variation, explore how specific vaginal bacterial communities are shaped by host factors, and provide a more comprehensive understanding of the role of vaginal microbiome in reproductive health among Chinese women.

## ASSOCIATIONS OF HOST FACTORS WITH THE VAGINAL MICROBIOME

A total of 7467 individuals were initially enrolled, of whom 6423 participants from 38 study centers across 18 provinces of China were included (Figure S1). All participants completed questionnaires, clinical examinations, and provided high‐quality vaginal microbiome data (Figure S2). Of these, 5666 (88.2%) from 32 centers were followed for 6 months, and 2838 (50.1%) had follow‐up examinations with documented clinical outcomes (mean follow‐up: 239 days) (Figure S1 and Table S1). This study has been registered online at ClinicalTrials.gov (NCT04694495) and China Human Genetic Resources Management Office (2021SLCJ0955). Detailed methods can be found in the supplementary methods.

Geographic variation in vaginal microbiota was evident (Figure S3 and Table S2), with mid‐latitude participants (30°–40° N) showing higher Shannon diversity than those from lower (20°–30° N) or higher (40°–50° N) latitudes, and diversity decreasing from western inland (90°–100° E) to eastern coastal regions (120°–130° E). At the vaginal community state types (CSTs) level, CST I was more prevalent in higher‐latitude and eastern regions, while CST IV‐B and Shannon diversity declined eastward. These patterns partly aligned with lower education levels and higher proportions of ethnic minorities in the West. Bray–Curtis analysis showed that microbiota dissimilarity increased with geographic distance, with lower within‐group than across‐group dissimilarity, indicating a role of geography in shaping composition.

**Figure 1:**
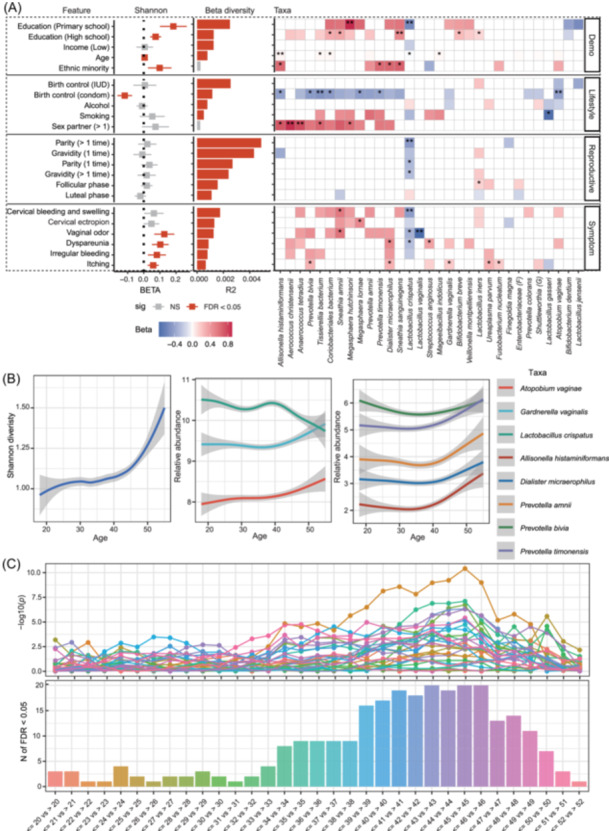
Associations between vaginal microbiome features and host factors. (A) Each panel displays associations of different levels of microbiome: beta diversity (by multivariate permutational multivariate analysis of variance (PERMANOVA) analysis, left), Shannon diversity (by linear regression, middle), and specific taxa (by linear regression, right). In the left and middle panels, R2 of PERMANOVA and the regression coefficient (beta) and standard error of linear regression analysis were plotted, and the color indicates the significance (gray, not significant, and red FDR < 0.05). In the right panel, the heatmap displaying the beta values of the linear regression analysis for each pair of association was plotted, and the color indicates the direction of association (red, positive, and blue, negative), the stars within each cell indicate the significance (*FDR < 0.1, **FDR < 0.05). Cells with color present nominal significant associations (*p* < 0.05) and blank cells represent nonsignificant associations. Detailed results of this figure are provided in Tables S3–S5. Only features with significant results in all three panels were plotted. IUD: intrauterine device. (B) Age‐related changes of bacterial species which were significant in the GAM analysis (FDR of gam < 0.05, and FDR of ANOVA comparing gam to linear model < 0.1). (C) The most significant difference in abundance of all tested taxa by a sliding window *t*‐test. The upper panel, −log10 of the *t*‐test *p*‐value comparing the mean of relative abundance of each bacterium before versus after this age; lower panel, number of significant taxa in each comparison with FDR < 0.05. Detailed information is provided in Tables S10–S12.

We assessed the associations between host features and vaginal microbiome characteristics, including beta diversity, Shannon diversity, and the abundance of 31 core species. A total of 82 associations were identified with a false discovery rate (FDR) < 0.1, and 226 associations with a *p*‐value < 0.05 (Figure 1 and Tables S3–S5). Symptoms showed the strongest associations, followed by demographics, and lifestyles. Top features with the greatest number of associations included condom use, age, number of sex partners, vaginal itching symptoms, and middle education level.

Condom use was associated with lower Shannon diversity and higher abundance of *Atopobium. vaginae* and *Prevotella. bivia*, while multiple sexual partners were linked to similar taxa with opposite effects without affecting overall diversity. Both factors showed no association with health‐associated *Lactobacillus* species. Low‐education participants were enriched with *Megasphaera. hutchinsoni*, *Sneathia. sanguinegens*, and *Lactobacillus. iners*, whereas *Lactobacillus. crispatus* was more abundant in higher‐education groups. Minority ethnic women had greater Shannon diversity and higher levels of dysbiosis‐associated taxa (e.g., *Prevotella. timonensis, Sneathia. sanguinegens*) than Han women, which may be partly influenced by distinct cultural practices [8], underscoring the need for targeted research and interventions in these populations.

Parity and gravidity explained the greatest variance in beta diversity but were unrelated to Shannon diversity, with *Lactobacillus. crispatus* consistently reduced in women with any reproductive history regardless of frequency or age. Self‐reported symptoms—particularly itching—were linked to higher alpha diversity and increase in taxa such as *Gardnerella. vaginalis, Ureaplasma. parvum*, and *Prevotella. bivia*, which can trigger pro‐inflammatory cytokines, mucosal irritation, and epithelial disruption [9, 10], potentially contributing to the observed symptoms such as itching.

*Lactobacillus. crispatus*, a key vaginal microbiome species, is strongly associated with multiple host features and negatively linked to health issues such as dyspareunia, cervical bleeding, and vaginal odor. Its abundance declines with age and reproductive history, suggesting potential benefits of supplementation for premenopausal women or those with reproductive experience. In contrast, *Lactobacillus. iners* is more prevalent and stable, shows fewer host associations, and may play a less protective role. Genomic comparisons reveal that *Lactobacillus. crispatus* carries more host‐beneficial genes, including d‐lactate dehydrogenase and mucin‐binding proteins, promoting pathogen resistance and anti‐inflammatory effects, whereas *Lactobacillus. iners* lacks d‐lactate production and is associated with activation of pro‐inflammatory pathways, suggesting its less protective role [11, 12]. While *Gardnerella. vaginalis* was associated solely with itching, other dysbiosis‐related species (*Sneathia. amnii, Dialister. micraerophilus, Streptococcus. anginosus*) were linked to multiple symptoms and host features, suggesting potential pathogenic roles.

Moreover, genus‐level analysis revealed 54 significant associations (FDR < 0.1) and 146 nominal associations (*p* < 0.05) (Table S6). Sensitivity analyses adjusting for STIs and library size produced consistent results, supporting the robustness of the findings (Table S7).

We further quantified variance explained by different host factor layers (Figure S4 and Tables S8, S9). Symptoms accounted for the largest proportion of variance (*R* ^2^ = 0.51%), followed by lifestyle (*R* ^2^ = 0.46%) and demographics (*R* ^2^ = 0.34%), but all combined explained only 1.62%—much lower than other body sites—indicating overall stability. Neutral model analysis (*R* ^2^ = 0.89; migration rate = 7E‐4, Figure S5) further supported the dominance of neutral processes. Variance explained varied widely by taxon (e.g., 7.45% for *Enterobacteriaceae* vs. 0.95% for *Lactobacillus. vaginalis*) and varied across different host layers, suggesting that different dysbiosis‐ and health‐associated bacteria are influenced by distinct host factors, warranting tailored clinical strategies.

## SIGNIFICANT SHIFT IN THE VAGINAL MICROBIOTA OCCURS AROUND 40 YEARS OLD

Aging is a well‐known driver of changes in the human microbiome [13]. Linear regression models revealed that age was significantly associated with alpha diversity and five taxa (Figure 1A). CST composition also varied by age: the prevalence of CST I significantly decreased after age 40, while CST IV‐B increased (Figure S6A). The generalized additive model (GAM) further revealed a nonlinear increase in Shannon diversity, with a marked increase around 40 years old (Figure 1B and Table S10), which was supported by a sliding window *t*‐test (Figure S6B and Table S11).

Eight species (25.8% of all the tested taxa) also presented significant nonlinear associations with age (FDR.GAM < 0.1 and FDR.Anova < 0.1, Figure 1B and Table S12), with a notable shift around age 40. The most significant taxa included *Lactobacillus. crispatus* and *Allisonella. histaminiformans* (also found in linear models), and *Gardnerella. vaginalis, Prevotella. bivia* and *Dialister. micraerophilus* (nonsignificant in linear models). A sliding window t‐test further confirmed that most changes began around age 40 (Figure 1C and Table S12). To further examine the age‐related bacterial transitions, participants were grouped as ≤40 or >40 years. We identified that *Lactobacillus. crispatus, Lactobacillus. gasseri*, and *Lactobacillus. jensenii* significantly decreased in women over 40, while dysbiosis‐related bacteria including *Prevotella. amnii, Atopobium. vaginae*, and *Gardnerella. vaginalis* significantly increased (Figure S6C). After adjustment for potential confounders, several key age‐associated taxa remained significant, while associations for some taxa disappeared after adjusting for menstrual cycle, likely due to hormonal influences or reduced power (Table S10). Mediation analysis further showed that lifestyle factors did not mediate age‐related changes in Shannon diversity or taxon abundance (Table S13).

Our study reveals a marked shift in the vaginal microbiome beginning around age 40, before the typical onset of menopause, characterized by a decline in *Lactobacillus. crispatus* and enrichment of dysbiosis‐associated taxa, independent of sociodemographic or behavioral factors. These changes likely reflect aging‐related hormonal alterations [14, 15] and other physiological transitions including vaginal pH [16], parity history, mucosal immunity [17, 18], and other molecular, such as circulating proteins and metabolites, aligning with evidence that the early 40s represent a critical window for molecular and microbiome remodeling [19]. Targeted interventions during this period may help preserve vaginal health and prevent dysbiosis.

## A MICROBIOME BALANCE SCORE INDICATING VAGINAL HEALTH

**Figure 2:**
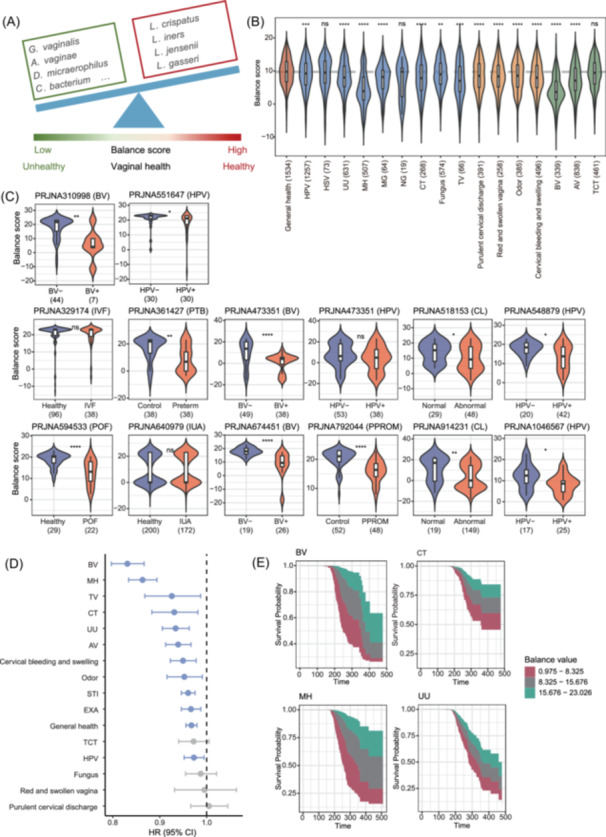
Vaginal microbiome balance score indicates health outcomes. (A) The definition of vaginal microbiome balance score. (B) Violin plots for balance score among individuals with different sexually transmitted infections, clinical symptoms, and vaginal or cervical diseases, comparing with those without any reported above phenotypes. Stars above the box indicate the significance which was obtained by Wilcoxon rank‐sum test. (C) Extrapolation of balance score (genus) to 13 vaginal microbiome case‐control studies. For each data set, the balance score (genus) between disease and control is shown. *p*‐value was obtained by Wilcoxon rank‐sum test. ****p < 1e‐4, ***p < 0.001, **p < 0.01, *p < 0.05, NS, not significant. (D) Associations between baseline balance score and follow‐up health outcomes. Hazard ratios (HRs) with 95% confidence intervals (CIs) and *p* values are shown from Cox regression models. Significant associations (FDR < 0.05) are highlighted in blue; nonsignificant results are shown in gray. (E) Survival plots for top features with the strongest associations in Cox regression. The x‐axis represents time in days. The area under the curve represents the probability of the outcome over time, with color gradients indicating values of the continuous balance score. Detailed information is provided Tables S14, S17, S19. A. vaginae = Atopobium. vaginae; AV = aerobic vaginitis; BV = bacterial vaginosis; C. bacterium = Coriobacteriales. bacterium; CT = Chlamydia trachomatis; D. micraerophilus = Dialister. micraerophilus; G. vaginalis = Gardnerella. Vaginalis; HPV = Human papillomavirus; HSV = Herpes simplex virus II; L. crispatus = Lactobacillus. crispatus; L. iners = Lactobacillus. iners; L. jensenii = Lactobacillus. jensenii; L. gasseri = Lactobacillus. gasseri; MH = Mycoplasma hominis; MG = Mycoplasma genitalium; NG = Neisseria gonorrhoeae; TV = Trichomonas vaginalis; TCT = cervical cytological results by ThinPrep Test; UU = Ureaplasma urealyticum.

Most differential bacteria showed consistent links to clinical symptoms and vaginal pathogens, enabling the definition of a healthy vaginal microbiome signature. Using a log ratio of healthy‐enriched to healthy‐depleted taxa, we developed a vaginal microbiome balance score (BS) (Figure 2A). Logistic regression using general health status (defined as absence of STIs, symptoms, bacterial vaginosis (BV), aerobic vaginitis (AV), and with normal cervical cytology) as the outcome identified species and genera enriched in healthy and unhealthy women, respectively (FDR < 0.05, Figure S7A,B and Table S14). Both genus‐ and species‐level BSs were significantly lower in unhealthy individuals, including those with STIs, symptoms, or vaginal diseases (Figure 2B, Figure S7C). Both scores were lower in women from ethnic minorities, smokers, condom users, those with larger waistlines, lower education, older age, and certain symptoms (Table S15). The species‐level BS mediated the effects of lifestyles: condom use and nonsmoking reduced dysbiosis risk by increasing the score (*p* < 0.05; mediation 15.3% and 24.3%, Figure S7E,F and Table S16). A marked drop in the score occurred around age 40, consistent with GAM analysis (Figure S7D). To evaluate the reproducibility of the BS, we validated our findings in 13 publicly available vaginal microbiome datasets on women's reproductive health (Table S17). Both scores can be replicated in most datasets (*p* < 0.05, Figure 2C, Figure S7G).

We further examined whether the baseline BS predicted the progression of vaginal health outcomes using longitudinal data. Participants who completed follow‐up were more likely to have higher education, but baseline prevalence of most clinical outcomes was similar to those lost to follow‐up (Table S18). A higher BS was significantly associated with a lower risk of BV, *Mycoplasma hominis* (MH), *Trichomonas vaginalis* (TV), and *Chlamydia trachomatis* (CT) at follow‐up (Figure 2D,E, Figure S8A, and Table S19), with results remaining robust after adjusting for baseline disease status (Figure S8B and Table S19). Stratified analyses showed that in the “new onset” group (outcome‐negative at baseline), a higher score predicted reduced risk of incident BV, AV, and vaginal odor, whereas in the “persistence” group (outcome‐positive at baseline), it was linked to lower persistence of *Ureaplasma urealyticum* (UU) (Figure S8D and Table S19). Sensitivity analysis among generally healthy women at baseline (*N* = 850) confirmed these associations, showing that higher scores were linked to reduced risk of AV and TV during follow‐up (Figure S8C and Table S19).

We next developed a prediction model using the baseline BS, training on baseline data and validating on follow‐up data. The model showed strong predictive power for future disease risk, particularly for BV (median AUC = 0.89 in training, 0.72 in testing), MH (0.83 in training, 0.70 in testing), and CT (0.72 in training, 0.67 in testing) (Figures S9, S10). While external validation is needed, these results highlight the BS's potential as a predictive tool for disease progression, especially for BV and STIs. Unlike conventional microscopic scoring systems such as the Nugent Score, which rely on subjective interpretation of Gram‐stained slides, the BS uses microbial abundances to provide an objective, biologically interpretable measure of vaginal health.

Our results are consistent with previous large‐scale vaginal microbiome studies (Table S20), showing age‐related increases in Shannon diversity, a decline in *Lactobacillus. crispatus*, and enrichment of dysbiosis‐associated taxa such as *Gardnerella. vaginalis* and *Prevotella. bivia*. Associations with education and multiple sexual partners were also confirmed, highlighting the robustness of these patterns. By analyzing a geographically and ethnically diverse Chinese population and introducing a validated microbiome BS, our study extends prior work and enhances the translational relevance of vaginal microbiome profiling, while some discrepancies with earlier studies may reflect population, sample size, or behavioral differences.

This study's major strengths include its large, multi‐center, national‐scale design, encompassing diverse regions across China, which enabled a comprehensive assessment of vaginal microbiota and host phenotypes. The clinically collected data on STIs and symptoms further strengthened the analyses. Limitations include the lack of hormonal measurements, preventing full separation of age‐versus hormone‐related microbial changes, and the observational design, which precludes causal inference between host factors and microbiome alterations. Self‐reported lifestyle and menstrual data may introduce recall bias, and differences in bioinformatics pipelines between discovery and validation datasets could have affected balance score results.

## AUTHOR CONTRIBUTIONS

**Cancan Qi**: Methodology; investigation; writing—original draft; visualization; writing—review and editing; formal analysis. **Yingxuan Zhang**: Investigation; Methodology; data curation; writing—review and editing. **Wei Qing**: Software; methodology; investigation; writing—review and editing. **Rongdan Chen**: Validation; writing—review and editing. **Zuyi Zhou**: Writing—review and editing; data curation. **Yumei Liu**: Writing—review and editing; data curation. **Enzhong Chen**: Writing—review and editing; data curation. **Wenyi Chen**: Writing—review and editing; validation. **Hongwei Zhou**: Conceptualization; funding acquisition; supervision; writing—review and editing; writing—original draft. **Muxuan Chen**: Conceptualization; funding acquisition; writing—original draft; writing—review and editing; supervision. All authors have read the final manuscript and approved it for publication.

## CONFLICT OF INTEREST STATEMENT

The authors declare no conflicts of interest.

## FUNDING INFORMATION

Basic and Applied Basic Research Foundation of Guangdong Province, Grant/Award Number: 2021B1515230007; National Natural Science Foundation of China, Grant/Award Numbers: NSFC 82002201, NSFC 82302610, NSFC81925026.

## ETHICS STATEMENT

All participants provided written informed consent, and the research has been approved by the Medical Ethics Committee of ZhuJiang Hospital (No. 2020‐KY‐071‐01).

## Supporting information


**Figure S1.** Study design and characteristics of this study.
**Figure S2.** Quality control of 16S sequencing data.
**Figure S3.** Geographical variation of the vaginal microbiota composition.
**Figure S4.** Variance explained by each layer of host factors on vaginal microbiome.
**Figure S5.** Neutral model and variance explained by different types of features on vaginal microbiome.
**Figure S6.** Association between age and vaginal microbiota.
**Figure S7.** Vaginal microbiome balance score and its associations with health outcomes at baseline.
**Figure S8.** Vaginal microbiome balance score and its associations with health outcomes at baseline.
**Figure S9.** Baseline microbiome balance score predicts future vaginal health outcomes.
**Figure S10.** AUC distributions for models predicting health outcomes at follow‐up.

**Table S1.** Characteristics of all samples in this study.
**Table S2.** Shannon diversity variations across different regions in China.
**Table S3.** Associations between each factor and Shannon diversity by linear regression model.
**Table S4.** Proportion of variance in microbiome composition that can be explained by each host factor by adonis analysis.
**Table S5.** Associations between each factor and relative abundance of taxa (at species level) by linear regression model.
**Table S6.** Associations between each factor and relative abundance of taxa (at genus level) by linear regression model.
**Table S7.** Associations between each factor and relative abundance of each bacterium additionally adjust for STI at both species and genus level.
**Table S8.** Variance explained by each feature layer on the relative abundance of each bacterium.
**Table S9.** Feature selected by 100 models in the analysis of variance explained.
**Table S10.** The non‐linear association between relative abundance of vaginal taxa and age by GAM model.
**Table S11.** Age of the most significant differences in Shannon diversity determined by a sliding window t‐test.
**Table S12.** Age of the most significant differences in taxa abundance determined by a sliding window t‐test.
**Table S13.** Mediation analysis assessing the role of vaginal microbiome in the association between age and lifestyles/symptoms (age, center and BMI were adjusted in the analysis).
**Table S14.** Bacteria (species and genera) associated with general health.
**Table S15.** Association between balance score and host features by linear regression model.
**Table S16.** Bi‐directional mediation analysis assessing the role of vaginal microbiome in the association between demographic/lifestyle features and balance score (age, center and BMI were adjusted in the analysis).
**Table S17.** Dataset used for replication of balance score.
**Table S18.** Baseline characteristics by follow‐up status.
**Table S19.** Baseline balance score and health outcomes in longitudinal study.
**Table S20.** Comparison between results from this study and other published studies.

## Data Availability

The full summary statistics to support the findings of this study are included within the supplementary information files. The microbiome sequencing data and limited metadata generated in this study have been deposited in the Genome Sequence Archive under study accession ID: HRA010936 (https://ngdc.cncb.ac.cn/gsa-human/browse/HRA010936). Due to ethical and legal restrictions, deidentified individual participant data and the data dictionary cannot be made publicly available. All data are available upon request to the corresponding author and subject to local rules and regulations. The data used for the main figures and scripts used in this study can be found on GitHub: https://github.com/ZJJY-Bioinformatics/CALM2004_landscape/. Supplementary materials (figures, methods, tables, graphical abstract, slides, videos, Chinese translated version, and update materials) may be found in the online DOI or iMeta Science http://www.imeta.science/.
