# Supplementary material for: Nationwide profiling of vaginal microbiota in Chinese women reveals age‐dependent shifts and predictive biomarkers for reproductive health
Source: Imeta. 2025 Oct 23;4(6):e70088. doi: 10.1002/imt2.70088 (PMC12747552; doi:10.1002/imt2.70088)
Supplement: Supplementary file 1 — Figure S1. Study design and characteristics of this study. Figure S2. Quality control of 16S sequencing data. Figure S3. Geographical variation of the vaginal microbiota composition. Figure S4. Variance explained by each layer of host factors on vaginal microbiome. Figure S5. Neutral model and variance explained by different types of features on vaginal microbiome. Figure S6. Association between age and vaginal microbiota. Figure S7. Vaginal microbiome balance score and its associations with health outcomes at baseline. Figure S8. Vaginal microbiome balance score and its associations with health outcomes at baseline. Figure S9. Baseline microbiome balance score predicts future vaginal health outcomes. Figure S10. AUC distributions for models predicting health outcomes at follow‐up. [file IMT2-4-e70088-s002.docx]

Supporting information to

**A large-scale characterization of the vaginal microbiota in over 6000 reproductive age women reveals key host-factor associations**

**Running title:** Large-scale study of vaginal microbiota and host factors

Cancan Qi^1,2^, Yingxuan Zhang^1,2^, Wei Qing^1,2^, Rongdan Chen^1,2^, Zuyi Zhou^1,2^, Yumei Liu^1,2^, Enzhong Chen^1,2^, Wenyi Chen^1,2^, CALM2004 Consortium Investigators^4^, Hongwei Zhou^1,2,3,5*^, Muxuan Chen^1,2,6*^

^1^Microbiome Medicine Center, Department of Laboratory Medicine, ZhuJiang Hospital, Southern Medical University, Guangzhou 510280, China

^2^Guangdong Provincial Clinical Research Center for Laboratory Medicine, Guangzhou 510280, China

^3^Department of Obstetrics and Gynaecology, Shenzhen Hospital, Southern Medical University, Shenzhen 518100, China

^4^Investigators listed in Supplementary materials

^5^State Key Laboratory of Organ Failure Research, Southern Medical University, Guangzhou 510515, China

^6^Department of Laboratory Medicine, Shenzhen Eye Hospital, Shenzhen Eye Medical Center, Southern Medical University, Shenzhen 518040, China

^*^Correspondence: [muxuanchen@126.com](mailto:muxuanchen@126.com) (Muxuan Chen); [hzhou@smu.edu.cn](mailto:hzhou@smu.edu.cn) (Hongwei Zhou)

**Supplementary methods**

**Study design and participants**

This observational study was performed within Chinese Association for Clinical Microbiome 2004 (CALM) project, a nationwide multi-center cohort study in China. Briefly, 7,467 premenopausal women were recruited from 41 study centers representing most provinces in China. After applying exclusion criteria, 28 women aged over 55 years, 275 women with a history of cervical tumors or surgery, and 331 participants without eligible vaginal samples were excluded. Additionally, 278 women without basic demographic information or clinical records were excluded from the analysis. Following quality control of the 16S sequencing data, 132 samples were removed due to sequencing failure or insufficient sequence depth (total reads < 1000) (Supplementary Figure.S1a). Study protocols including participant recruitment, and sample collection, processing and testing have been reported previously [1]. This study has been registered online at ClinicalTrials.gov (NCT04694495) and China Human Genetic Resources Management Office (2021SLCJ0955). All participants provided written informed consent and the research has been approved by the Medical Ethics Committee of ZhuJiang Hospital (No. 2020-KY-071-01).

**Follow-up**

Participants from 32 centres were followed up at around six months. Vaginal and cervical samples were collected with the sample protocol during both baseline and the follow-up visit (between May 2021 and June 2023). Clinical examination and host features were recorded at both baseline and follow-up using the uniformed methodology below.

**Vaginal and cervical sample collection**

Participants underwent cervical screening at each study center following standardized protocols. During the screening, vaginal secretion and cervical exfoliated cell samples were collected. Vaginal secretion samples were obtained from the posterior fornix using a sterile disposable swab while participants were positioned in lithotomy. These samples were primarily used for assessment of vaginal microbiome. Cervical exfoliated cells were collected using a specialized cervical cytology brush (Hybribio, China) for the identification of human papillomavirus (HPV) types, detection of sexually transmitted infections (STIs), and the performance of ThinPrep Cytologic Tests (TCT). All sample collections were carried out by trained clinicians.

**Clinical assessments**

During the vaginal examination, clinicians assessed and documented cervical or vaginal symptoms including purulent cervical discharge, vaginal wall redness and swelling (Red and swollen vagina), vaginal odor (Odor), cervical bleeding and swelling, and cervical ectropion. In parallel, all participants completed a self-reported questionnaire regarding vaginal symptoms. The questionnaire included questions about the presence of vaginal itching (Itching), lower abdominal pain (Abnormal pain), painful urination or lumbago (Urodynia), pain or bleeding during sexual activity (Dyspareunia), irregular vaginal bleeding (Irregular bleeding), and symptoms of urinary urgency or frequency (Frequency/urgency of urination) (Figure.1a).

Demographics (age, BMI, waist circumference, and ethnicity), socio-economic (education and income level), lifestyle (smoking, alcohol consumption, number of sexual partners, sexual activity within 72 hours of clinical examination, and contraceptive use), reproductive (parity, gravidity, history of adverse pregnancy outcomes, menstrual irregularities, and current menstrual phase), medication history (Estrogen, glucose- and lipid-modulating drugs (Glulip), and antibiotics within the past month), and disease history (hypertension, diabetes, and urinary tract infections [UTIs]) were collected through a self-reported questionnaire administered during the clinical examination. Ethnicity was categorized as Han or ethnic minority. Education level was classified into three groups: college or higher (control), high school, and junior school. Income was classified as high (control), middle, or low. Birth control methods were grouped into four categories: none (control), intrauterine device (IUD), contraceptive pills (Pills), and condoms. The number of sexual partners was classified into two categories: one (control) and two or more. Gravidity, parity, and history of adverse pregnancy outcomes were divided into three groups: none (control), one, and two or more.

The menstrual cycle phase was determined based on the sampling date, the reported start date of the menstrual cycle, and the average cycle length. Using this information, the cycle day for each participant was calculated. The menstrual cycle was then categorized into three phases: follicular phase, ovulatory phase, and luteal phase, based on the corresponding cycle day.

Sexually transmitted pathogens, including *Neisseria gonorrhoeae*, *Mycoplasma hominis*, *Mycoplasma genitalium*, *Herpes simplex virus II* (HSV-2), *Chlamydia trachomatis*, and *Ureaplasma urealyticum*, were assessed using a STI detection kit (Hybribio, China) following the manufacturer’s instructions. Human papillomavirus (HPV) was tested for 21 subtypes (13 high-risk and 8 low-risk) using HPV GenoArray diagnostic kit (Hybribio, China). The presence of *Trichomonas vaginalis* was determined by wet mount microscopy. Fungal infections were detected using both wet mount microscopy and culture methods for vaginal secretions. Additionally, a binary STI phenotype was defined, where a positive case was characterized by a positive result for any of the above pathogens, and a negative case was defined by the absence of all the listed pathogens.

Cervical exfoliated cell samples were placed in ThinPrep Pap Test PreservCyt Solution (Hybribio, China) and further analysed using the Bethesda system (TBS) for the ThinPrep Test (TCT). The results were classified based on the TBS grading system, including negative for intraepithelial lesion or malignancy (NILM), atypical squamous cells (ASC), atypical glandular cells (AGC), low-grade squamous intraepithelial lesion (LSIL), high-grade squamous intraepithelial lesion (HSIL), and squamous cervical cancer (SCC). A categorical TCT variable was defined as 0 = NILM, and 1 = other grades.

**DNA extraction, amplification, sequencing and preprocessing**

Vaginal secretions were used for bacterial DNA extraction, using QIAamp DNA Micro Kit (QIAGEN, Germany) following the manufacturer’s instructions. To achieve high species-level resolution of vaginal microbiota, we applied an optimized taxonomic classification pipeline previously developed and validated by our group, which has demonstrated high accuracy in vaginal bacterial species identification [2]. The V1-V3 region of 16S rRNA gene was amplified by PCR and were sequenced on Illumina Novaseq PE250 platform. The raw sequencing data were demultiplexed into sample pair-end fastq files based on unique barcodes and trimmed by removing the barcodes and primer sequences using a customized Perl script to retain reads with a minimum length of 100 bp. The forward reads were denoised by DADA2 [3] (Version 2021.11), including quality filtering (--p-trunc-len 223), dereplication and chimeras filtering, and then the amplicon sequence variant (ASV) table and the representative sequences were generated. The taxonomic classification was performed following our pipeline determined optimal for the species-level classification of vaginal microbiota [2]: the representative sequences were annotated by QIIME2 using the pre-fitted scikit-learn taxonomy classifier [4] based on the combination of Greengenes2 [5] (Release 2022.10), SILVA [6] (Version 138) and RDP [7] (Release 11.5).

Rarefaction was applied to determine the minimum sequencing depth needed for stable diversity estimation, and samples with low sequence depth (total reads < 1000) were excluded from the downstream analyses. To control for potential contamination, we included 190 blank swab controls (approximately five per study center) and 9 DNA extraction buffer controls. Sequencing yielded usable data for 163 blank swabs and all 9 buffer controls. Total read counts across all controls and found that the vast majority fell below the threshold of 1,000 reads, as defined by rarefaction-based cut-off for downstream inclusion (Figure S2). Decontamination was assessed using the r package decontam [8], combined with manual inspection of putative contaminating ASVs, according to the bacterial density and frequency, as well as the black list that reported known reagent and laboratory contamination [9,10].

**Statistical analysis**

Association between vaginal microbiota features and host features

Pairwise spearman correlation analysis was performed to assess the association between each pair of tested host features. The microbial community composition (beta diversity) was assessed using Bray-Curtis dissimilarity, based on the relative abundance of microbial species. The proportion of variance in microbiome composition attributable to each host feature was determined using permutational multivariate analysis of variance (PERMANOVA), with the adonis2 function from the R package "vegan" (1,000 permutations). This analysis adjusted for study center, age, and BMI. For analyses involving age and BMI, adjustments were made only for study center. To evaluate the combined effect of host features, a multivariate PERMANOVA was performed, incorporating all factors that showed significant associations (FDR < 0.05) in the univariate analysis [11]. Shannon diversity index was calculated using the "microbiome" R package. The association between Shannon diversity index and each host factor was evaluated using linear regression models, adjusting for the same variables as in the PERMANOVA analysis (age, BMI and study center).

Linear regression models were applied to examine the associations between host features and the relative abundance of each vaginal microbiota taxon at the species level, including 31 core species present in at least 10% of the samples. The relative abundance (RA) of each taxon was transformed using the centered log-ratio (CLR) transformation for association analysis. The same linear regression model used for Shannon diversity index was employed to assess the relationship between each host feature and individual species, including age, BMI and study center as covariates. Given the potential impact of unbalanced data on the statistical power (59.5% of features had a prevalence < 20%), the results of all association analyses were considered significant at FDR of < 0.1, adjusted using the Benjamini-Hochberg method. To account for the potential influence of sexually transmitted infections (STIs) on the vaginal microbiota, as reported in previous studies, STI status was included as an additional covariate in a sensitivity analysis. Community state type (CST) was calculated using the VALENCIA (VAginaL community state typE Nearest CentroId clAssifier) method [12]. The associations between each CST (compared to other types) and host features were assessed using logistic regression model adjusting for age, BMI and study center.

Non-linear relationship between age and vaginal microbiota

Two additional non-linear analysis models were applied to further investigate the changes in vaginal microbiota features with aging. First, a generalized additive model (GAM) with integrated smoothness estimation was fitted using the mgcv package in R, as described in previous studies [13]. The model was specified as follows: CLR-transformed relative abundance (RA) of each bacterium ~ s(age) + BMI + study center, where s(age) represents a spline smooth of age. The estimated effect size was calculated using Cohen’s f², as previously reported [13]. The GAM was then compared with a linear model without the spline smooth of age using analysis of variance (ANOVA; "anova" function in R). Bacteria with an FDR < 0.1 in the s(age) term of the GAM, as well as those with FDR < 0.1 in the ANOVA analysis, were considered significantly associated with age in a non-linear manner.

To ensure robustness, several sensitivity analyses was conducted, adjusting for study center, BMI, education level, birth control measures, number of pregnancies (gravidity), and STI status. These covariates were selected based on their significant associations with both age and vaginal microbiota features, as determined by Spearman correlation (*p* < 0.05 in both associations, and a correlation coefficient > 0.1 in at least one association). Highly correlated variables were excluded to avoid multicollinearity. To account for the influence of hormone, we conducted a sensitivity analysis by adjusting for menstrual cycle phase as a proxy for hormonal fluctuations. Given the reduced sample size in this sensitivity analysis, bacteria with a raw *p* value < 0.05 were prioritized.

Second, a sliding-window t-test was applied to compare Shannon diversity index and bacterial abundance before and after each year of age. To ensure sufficient sample size in each age group, the analysis was limited to ages 22 through 52, as the youngest and oldest age groups had smaller sample sizes, and each group needed to include at least 30 samples.

Variance explained by each host factor on each vaginal bacterium

Variance of relative abundance of each bacterium explained by different types of host factors including demographics, lifestyle, reproductive features, clinical symptoms, drug history and disease history, as well as other external factors including study center and STIs, was calculated according to previous study [14]. The glmnet R package was applied to investigate the variance of bacteria that could be explained by the above host features using lasso regression while employing a 10-fold cross-validation. And then the independent percentage of explained variance (R^2^) was calculated for each host feature on each bacterium. The independent R^2^ of all host factors from the same layer that explained significant variance were summed up as the representative of the R^2^ explained by the layer. This procedure was repeated for 100 times to avoid the randomness of lasso selection, and the average R^2^ of each layer was calculated accordingly.

Mediation analysis

Mediation analysis was performed to infer the role of vaginal microbiota in the following associations:

1) whether demographics/ lifestyles/ reproductive features mediate the effect of aging on the vaginal microbiota change (shannon diversity index and individual bacterium);

2) whether vaginal microbiota balance score mediates the associations between demographics/ lifestyles and general vaginal/ cervical health. In this analysis, demographic or lifestyle was treated as the exposure, balance score was treated as either mediator in direction1 (lifestyle -> balance score -> general health) or as outcome in direction2 (lifestyle -> general health -> balance score).

In all the above analyses, microbial features associated with both exposures (demographics/ lifestyles/ reproductive features) and outcomes (vaginal/ cervical symptoms or general healthy status) was selected first, and the mediation analysis of these association groups were performed using R package mediation. Age, BMI and study center was adjusted in the model except for the analysis of age (analysis 1).

Calculation of vaginal microbiome balance score

The microbiota balance score, which was defined as a log ratio between the geometric means of healthy-enriched and unhealthy-enriched bacteria, was calculated according to Bjork et al [15]. In brief, let X = (X_1_, X_2_, X_3_, …, X_k_) be a sample with k features (here referring to microbial taxa). Given two non-overlapping subsets of features in X denoted by X_+_ and X_−_, indexed by I_+_ and I_−_, and comprising k_+_ (number of features enriched in X_+_) and k_−_ (number of features enriched in X_−_). The balance score between X_+_ (general healthy) and X_−_ (general unhealthy) is defined as the log ratio of the geometric mean of the two subsets of features as follows:

$$\text{B}\left( \text{X}_{\text{+}}\text{,}\text{X}_{\text{-}} \right)\text{ }\text{=}\text{ }\text{log(}\frac{{\text{((}\prod_{\text{i}\text{∈}\text{I}_{\text{+}}} \text{)}\text{X}_{\text{i}}\text{)}}^{\text{1/}\text{k}_{\text{+}}}}{{\text{((}\prod_{\text{i}\text{∈}\text{I}_{\text{-}}} \text{)}\text{X}_{\text{j}}\text{)}}^{\text{1/}\text{k}_{\text{-}}}}\text{)}$$

We can further simply the equation as:

$$\text{B}\left( \text{X}_{\text{+}}\text{,}\text{X}_{\text{-}} \right)\text{ }\text{=}\text{ }\frac{\text{1}}{\text{k}_{\text{+}}}\sum_{\text{i}\text{∈}\text{I}_{\text{+}}} \text{log}\text{X}_{\text{i}}\text{ - }\frac{\text{1}}{\text{k}_{\text{-}}}\sum_{\text{i}\text{∈}\text{I}_{\text{-}}} \text{log}\text{X}_{\text{j}}$$

Using the relative abundances of the vaginal microbiota taxa at species and genus level, we computed balance scores using the above formula according to Bjork et al in our dataset. The features were selected by logistic regression model with FDR < 0.05, and the model is General healthy ~ taxa + age + BMI + study center, where the general healthy group was defined as participant with normal TCT results, without vaginal/ cervical symptoms by clinical examination, BV, AV and without vaginal pathogen infections that were measured in this study. The pathogen infections included: Human papillomavirus (HPV), *Neisseria gonorrhoeae* (NG), *Chlamydia trachomatis* (CT), *Ureaplasma urealyticum* (UU), *Mycoplasma hominis* (MH), *Mycoplasma genitalium* (MG), *Herpes simplex* virus type *II* (HSV-2) and fungus. Associations between balance score and tested host factors were further assessed by linear regression model adjusting for age, study center and BMI.

To validate the balance score, we collected publicly available vaginal microbiome datasets generated by 16S sequencing by searching the GEO database. After filtering out studies that were not relevant to vaginal health, lack of healthy controls or metadata, 13 studies were retained. The raw sequencing data of each dataset were processed using a uniform analysis pipeline [2]. Most datasets targeted the V3–V4 region, differing from the V1–V3 region used here, which could limit species-level resolution as noted previously [2]. Therefore, we replicated the score at both genus and species levels. The balance score was calculated for each dataset using the same taxa list as predefined in our dataset. And the difference in the balance score between healthy and unhealthy groups was evaluated by Wilcoxon test according to the phenotype in each dataset.

Baseline microbiome balance score and disease progress

Cox regression models were used to evaluate the association between baseline vaginal microbiome balance scores and the progression of vaginal health outcomes. Two models were constructed for the primary analysis: (1) Follow-up outcome ~ balance score (Species) + age + BMI + study center, and (2) Follow-up outcome ~ balance score (Species) + age + BMI + study center + strata (baseline outcome). Model (2) included stratification by baseline disease status to control for initial differences in clinical condition. Additionally, a sensitivity analysis was conducted among women who were generally healthy at baseline, using the same covariate structure as model (1).

To further characterize disease progression, we defined two outcome trajectories based on baseline and follow-up status: Persistency (P): participants with disease present at both baseline and follow-up, compared to those who were positive at baseline but negative at follow-up. New-onset (N): participants who were negative at baseline and became positive at follow-up, compared to those who remained negative. These two progression outcomes were analyzed using the following models: (3) Outcome progress (new-onset) ~ baseline balance score + age + BMI + study center, and (4) Outcome progress (persistency) ~ baseline balance score + age + BMI + study center.

To assess the predictive utility of the baseline balance score, we built a machine learning model to predict follow-up disease outcomes. Gradient Boosting Machine (GBM) was employed, using baseline data as the training set and follow-up data as the testing set. Hyperparameters were tuned using 10-fold cross-validation, repeated five times. To mitigate randomness and ensure robust estimates, the entire training and evaluation process was repeated 100 times. After training in the baseline data, the models were further applied to the follow-up data. The median area under the receiver operating characteristic (ROC) curve (AUC) from the 100 models of testing set was used to represent overall model performance, and the corresponding ROC curve was plotted.

**References**

1. Zhou, Zuyi, Yi Hou, Wei Qing, Yiya Shi, Yingxuan Zhang, Rongdan Chen, Jinxia Ou, et al. 2023. “The association of hpv infection and vaginal microbiota of reproductive women in china: a multicenter cohort study protocol.” *Medicine in Microecology* 15: 100072. https://doi.org/10.1016/j.medmic.2022.100072

2. Qing, Wei, Yiya Shi, Rongdan Chen, Yin’ai Zou, Cancan Qi, Yingxuan Zhang, Zuyi Zhou, et al. 2024. “Species-level resolution for the vaginal microbiota with short amplicons.” *mSystems* e0103923. https://doi.org/10.1128/msystems.01039-23

3. Callahan, Benjamin J., Paul J. McMurdie, Michael J. Rosen, Andrew W. Han, Amy Jo A. Johnson, and Susan P. Holmes. 2016. “DADA2: high-resolution sample inference from illumina amplicon data.” *Nature Methods* 13: 581–583. https://doi.org/10.1038/nmeth.3869

4. Pedregosa, Fabian, Gaël Varoquaux, Alexandre Gramfort, Vincent Michel, Bertrand Thirion, Olivier Grisel, Mathieu Blondel, et al. 2011. “Scikit-learn: machine learning in python.” *The Journal of Machine Learning Research* 12: 2825–2830.

5. McDonald, Daniel, Yueyu Jiang, Metin Balaban, Kalen Cantrell, Qiyun Zhu, Antonio Gonzalez, James T. Morton, et al. 2022. *Greengenes2 enables a shared data universe for microbiome studies*. *preprint*. Bioinformatics. https://doi.org/10.1101/2022.12.19.520774

6. Quast, Christian, Elmar Pruesse, Pelin Yilmaz, Jan Gerken, Timmy Schweer, Pablo Yarza, Jörg Peplies, et al. 2013. “The silva ribosomal rna gene database project: improved data processing and web-based tools.” *Nucleic Acids Research* 41: D590-596. https://doi.org/10.1093/nar/gks1219

7. Cole, James R., Qiong Wang, Jordan A. Fish, Benli Chai, Donna M. McGarrell, Yanni Sun, C. Titus Brown, et al. 2014. “Ribosomal database project: data and tools for high throughput rrna analysis.” *Nucleic Acids Research* 42: D633-642. https://doi.org/10.1093/nar/gkt1244

8. Davis, Nicole M., Diana M. Proctor, Susan P. Holmes, David A. Relman, and Benjamin J. Callahan. 2018. “Simple statistical identification and removal of contaminant sequences in marker-gene and metagenomics data.” *Microbiome* 6: 226. https://doi.org/10.1186/s40168-018-0605-2

9. Callahan, Benjamin J., Daniel B. DiGiulio, Daniela S. Aliaga Goltsman, Christine L. Sun, Elizabeth K. Costello, Pratheepa Jeganathan, Joseph R. Biggio, et al. 2017. “Replication and refinement of a vaginal microbial signature of preterm birth in two racially distinct cohorts of us women.” *Proceedings of the National Academy of Sciences of the United States of America* 114: 9966–9971. https://doi.org/10.1073/pnas.1705899114

10. Salter, Susannah J., Michael J. Cox, Elena M. Turek, Szymon T. Calus, William O. Cookson, Miriam F. Moffatt, Paul Turner, et al. 2014. “Reagent and laboratory contamination can critically impact sequence-based microbiome analyses.” *BMC Biology* 12: 87. https://doi.org/10.1186/s12915-014-0087-z

11. Gacesa, R., A. Kurilshikov, A. Vich Vila, T. Sinha, M. A. Y. Klaassen, L. A. Bolte, S. Andreu-Sánchez, et al. 2022. “Environmental factors shaping the gut microbiome in a dutch population.” *Nature* 604: 732–739. https://doi.org/10.1038/s41586-022-04567-7

12. France, Michael T., Bing Ma, Pawel Gajer, Sarah Brown, Michael S. Humphrys, Johanna B. Holm, L. Elaine Waetjen, et al. 2020. “VALENCIA: a nearest centroid classification method for vaginal microbial communities based on composition.” *Microbiome* 8(1): 166. https://doi.org/10.1186/s40168-020-00934-6

13. Zhernakova, Daria V., Trishla Sinha, Sergio Andreu-Sánchez, Jelmer R. Prins, Alexander Kurilshikov, Jan-Willem Balder, Serena Sanna, et al. 2022. “Age-dependent sex differences in cardiometabolic risk factors.” *Nature Cardiovascular Research* 1: 844–854. https://doi.org/10.1038/s44161-022-00131-8

14. Hu, Shixian, Arno R. Bourgonje, Ranko Gacesa, Bernadien H. Jansen, Johannes R. Björk, Amber Bangma, Iwan J. Hidding, et al. 2024. “Mucosal host-microbe interactions associate with clinical phenotypes in inflammatory bowel disease.” *Nature Communications* 15: 1470. https://doi.org/10.1038/s41467-024-45855-2

15. Björk, Johannes R., Laura A. Bolte, Andrew Maltez Thomas, Karla A. Lee, Niccolo Rossi, Thijs T. Wind, Lotte M. Smit, et al. 2024. “Longitudinal gut microbiome changes in immune checkpoint blockade-treated advanced melanoma.” *Nature Medicine* 30: 785–796. https://doi.org/10.1038/s41591-024-02803-3


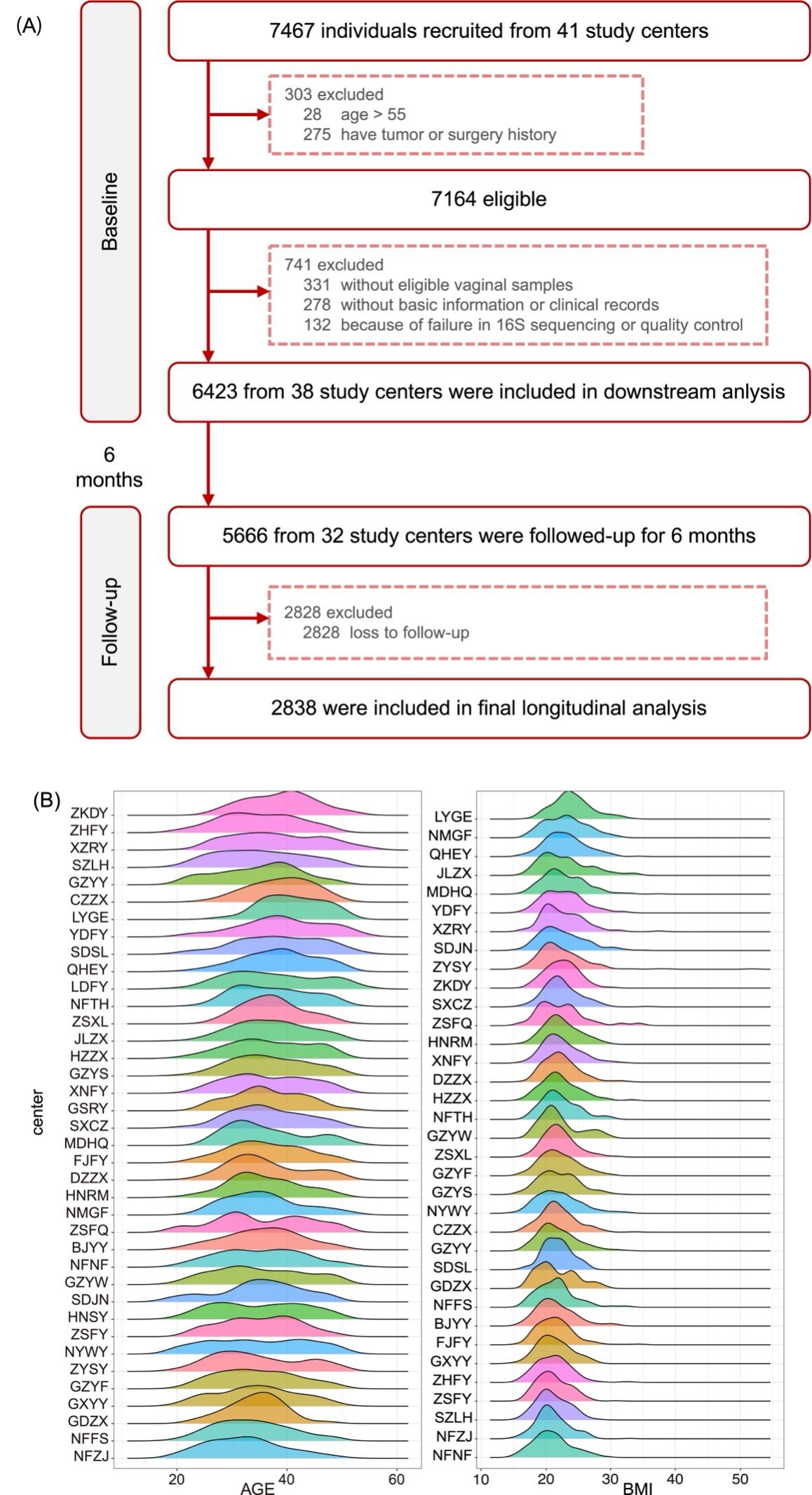


## Figure S1 Study design and characteristics of this study. (A) Exclusion criteria in the studied population. (B) Distribution of age and across different study centers.

**
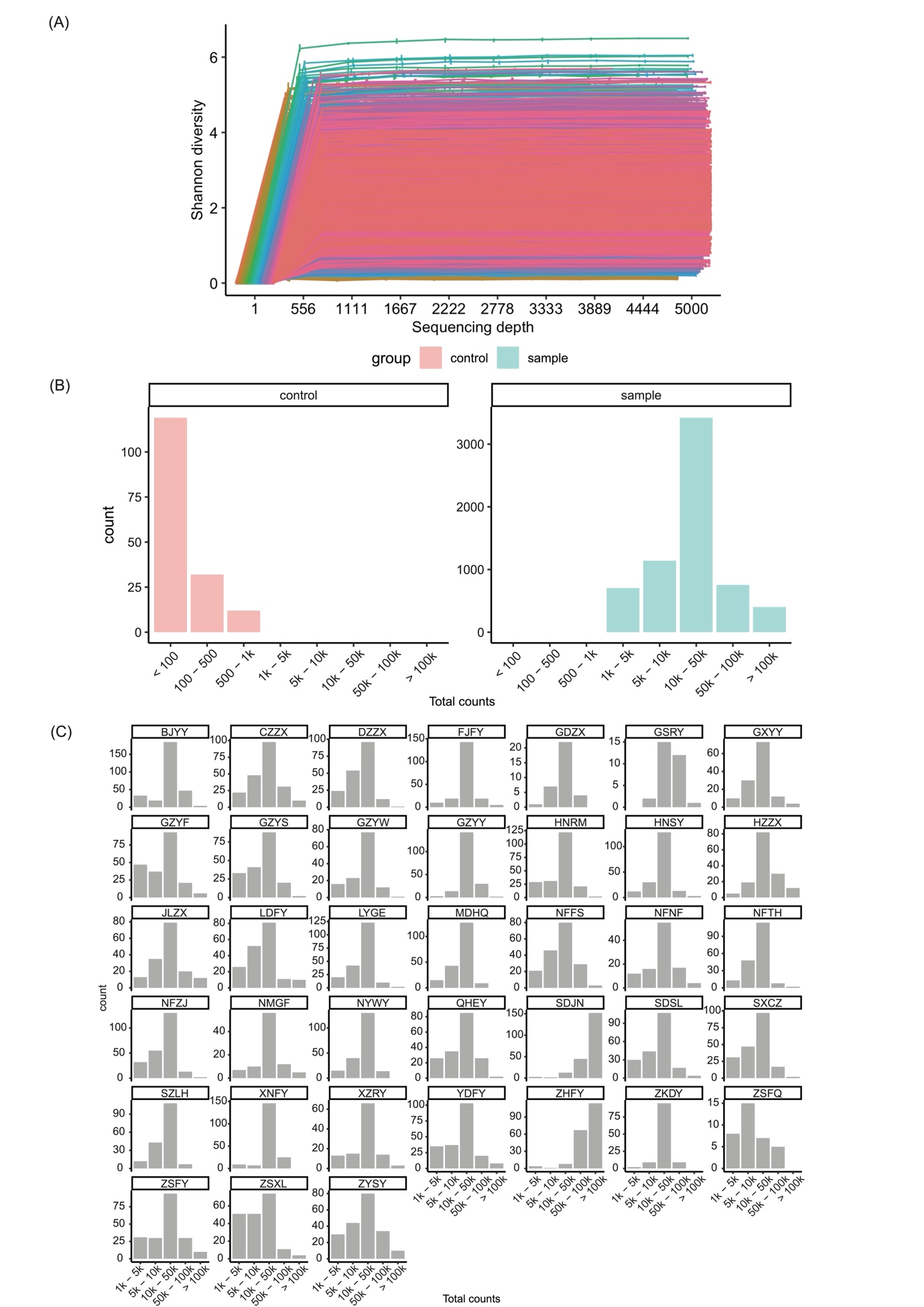
**

## Figure S2 Quality control of 16S sequencing data. (A) Rarefaction curve. (B) Distribution of total counts per sample including control samples (left) and real samples (right). (C) Distribution of library size across different study centers.


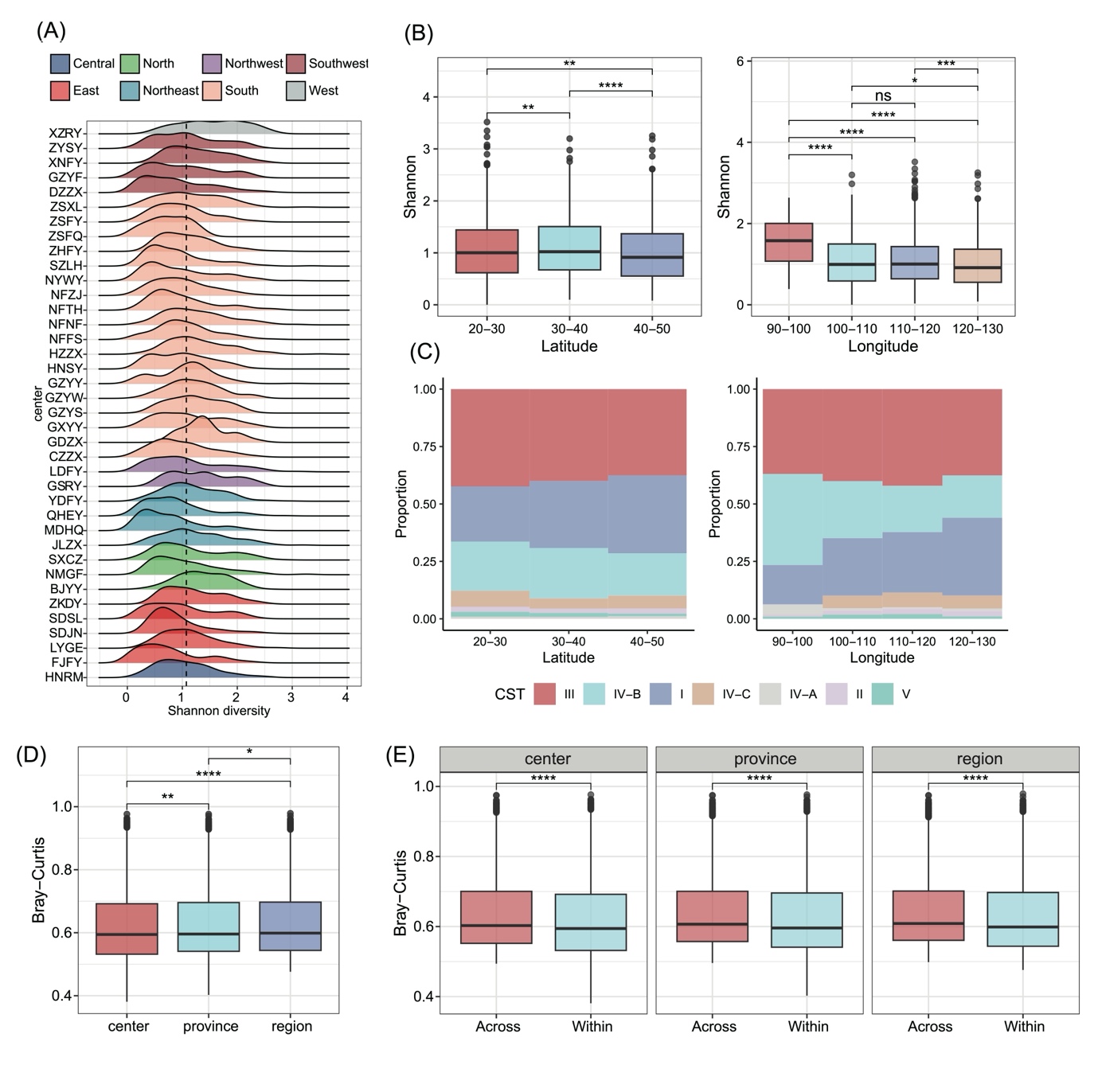


## Figure S3 Geographical variation of the vaginal microbiota composition. (A) Density plot illustrating the distribution of Shannon diversity across different study centers. (B) Boxplots showing the variation in Shannon diversity across different latitudes and longitudes. (C) Bar plot showing the proportion of Community State Type (CST) across different latitudes and longitudes. D. Boxplots illustrating increased pairwise Bray-Curtis dissimilarity among individuals residing within the same center, province and region. E. Boxplots visualizing pairwise Bray-Curtis dissimilarity among individuals within verse across different cetners, provinces and regions.


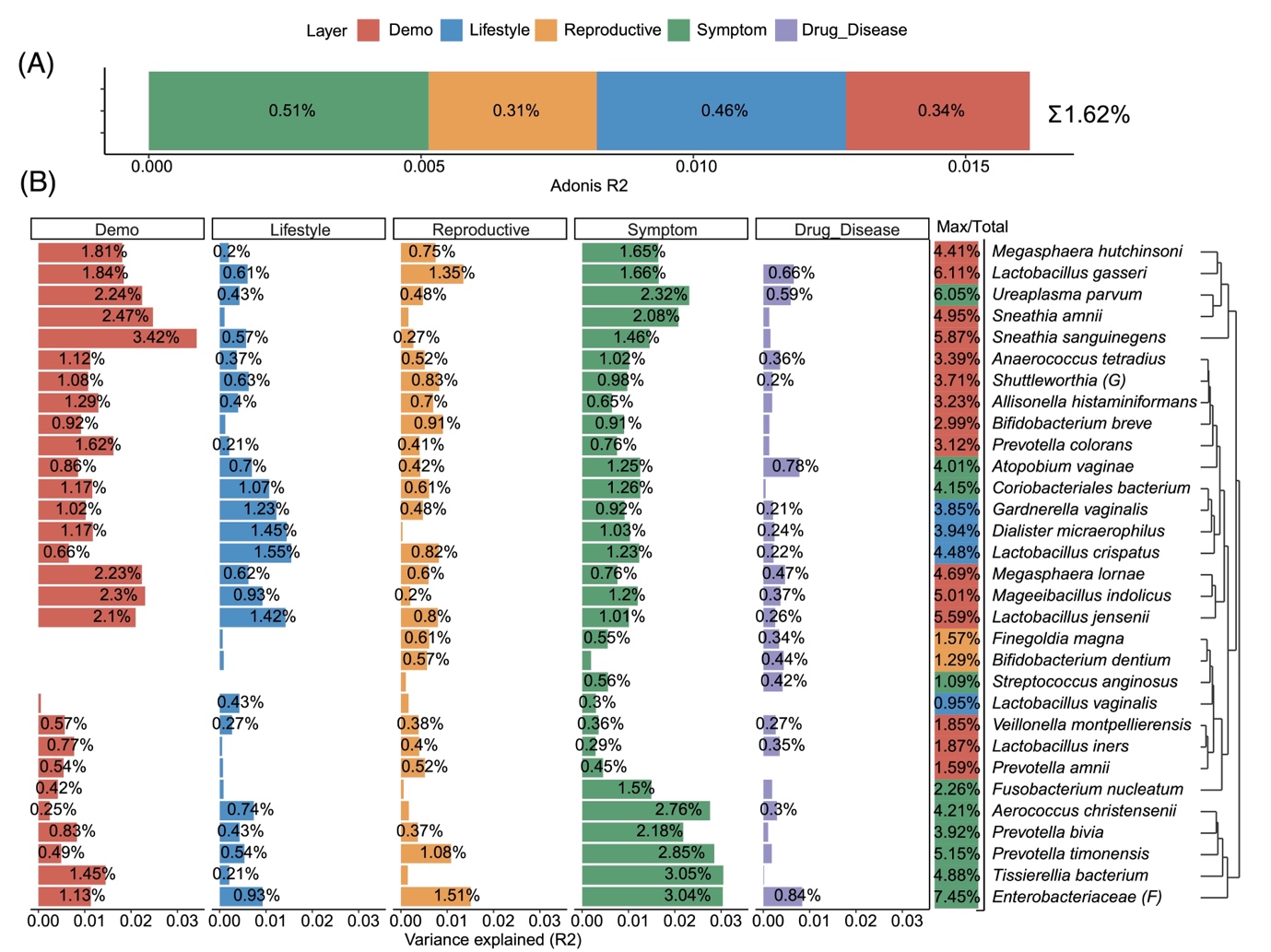


## Figure S4 Variance explained by each layer of host factors on vaginal microbiome. (A) Variance in microbiome composition explained by each layer of host factors by multivariate PERMANOVA analysis. (B) Bar plots showing the variance explained by each layer of host factors on the relative abundance of core taxa. Detailed information is provided in Table S8.


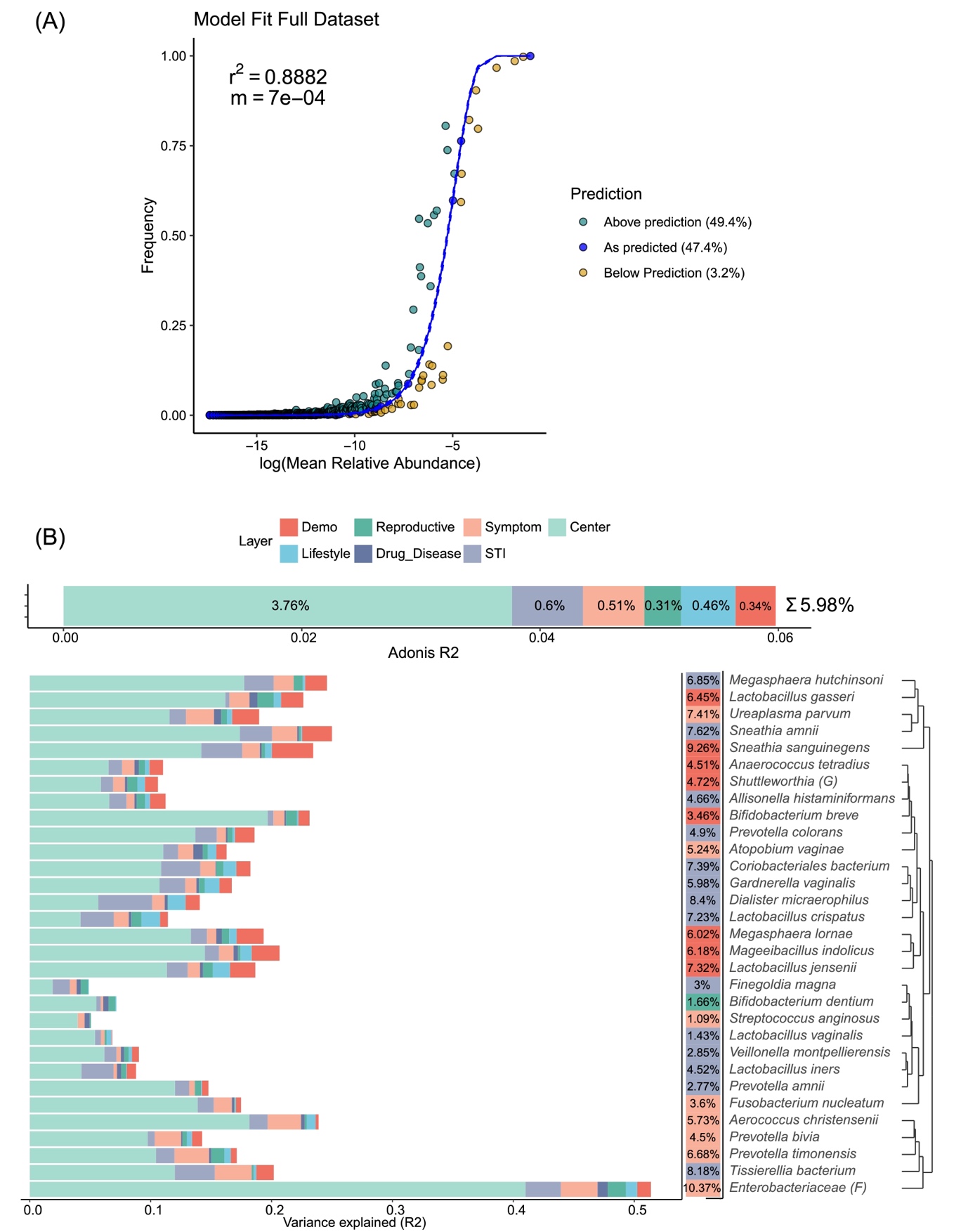


## Figure S5 Neutral model and variance explained by different types of features on vaginal microbiome. (A) Fit of the neutral community model (NCM) of community assembly. The solid blue lines indicate the best fit to the NCM, and the dashed blue lines represent 95% confidence intervals (CIs) around the model prediction. ASVs that occur more or less frequently than predicted by the NCM are shown in different colors. m indicates the immigration, R2 indicates the fit to this model. (B) Top: variance in microbiome composition explained by each layer of host factors by multivariate PERMANOVA analysis. Bottom: Bar plots showing the variance explained by each layer of features on the relative abundance of core taxa. Detailed information is provided in Table S8.


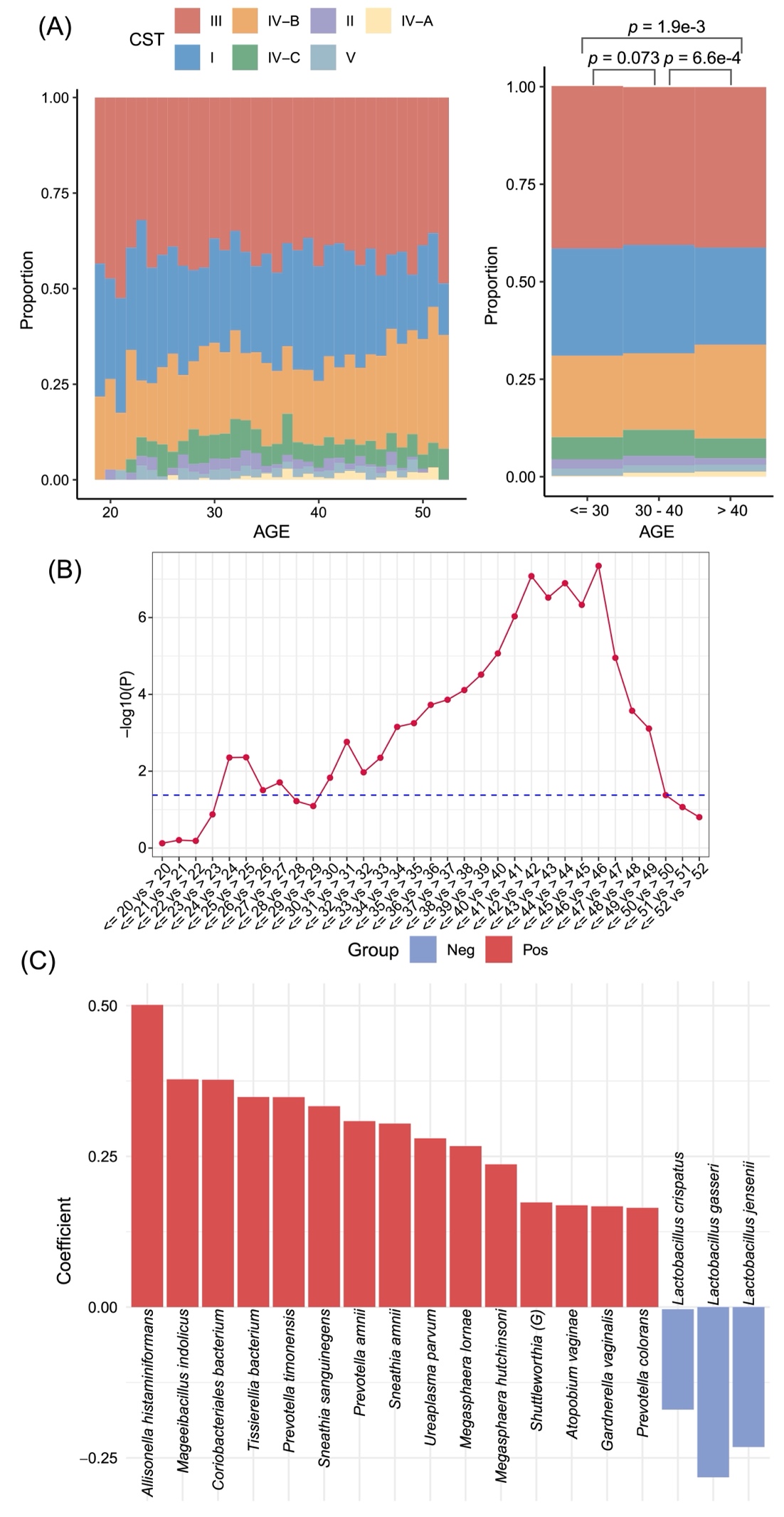


## Figure S6 Association between age and vaginal microbiota. (A) Bar plots showing the distribution of Community State Types (CSTs) across different age groups. The *p* value above the bar plot on the right indicates the overall group difference assessed by chi-square test. (B) The most significant difference in Shannon diversity by a sliding window t-test. For each year of age, dots present -log10 of the t-test *p* value comparing the mean of Shannon diversity before versus after this age. (C) Differentially abundant taxa between women aged ≤ 40 and > 40 years identified by linear regression. Taxa enriched in women over 40 are shown in red; those enriched in women 40 or younger are shown in blue.


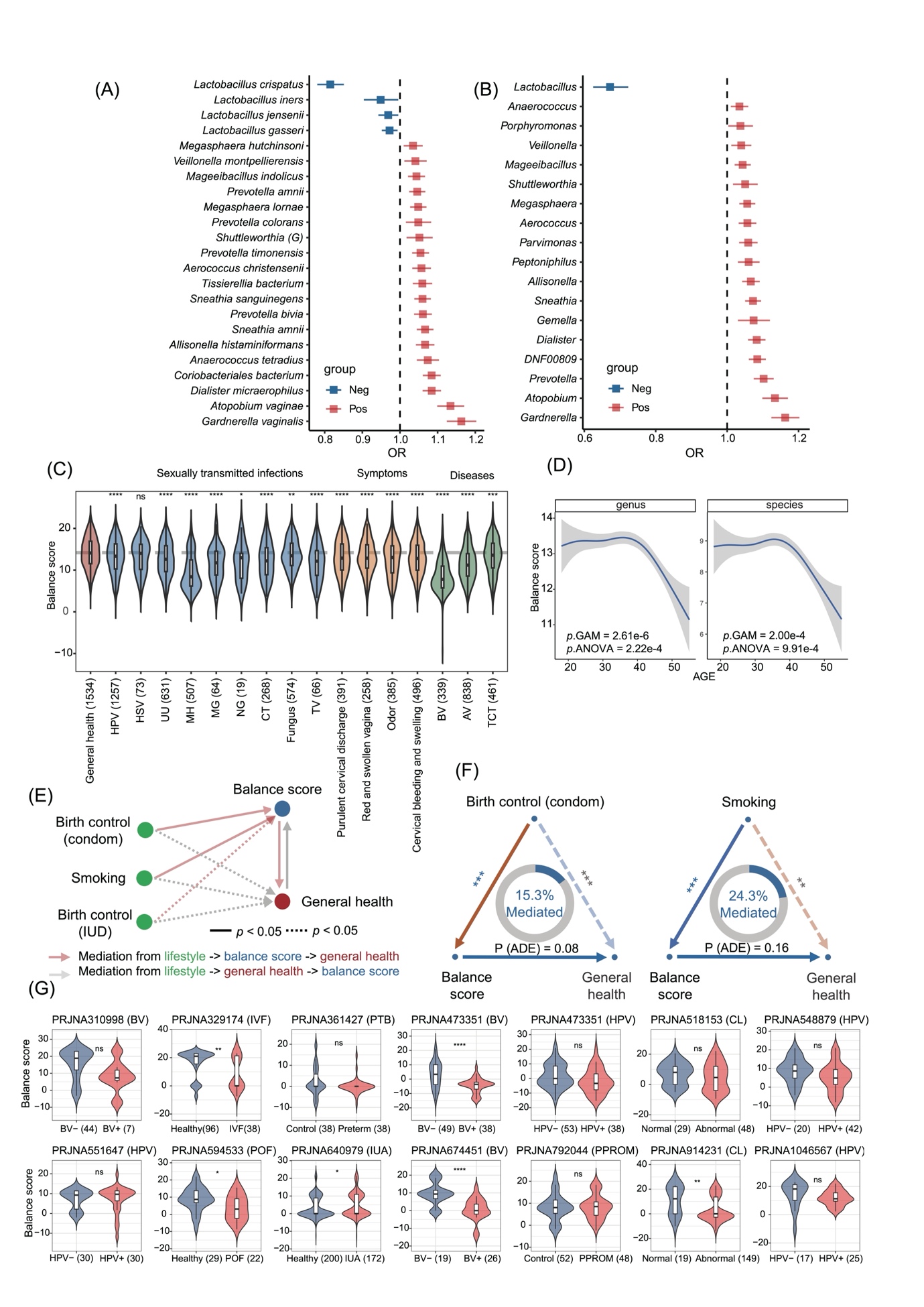


## Figure S7 Vaginal microbiome balance score and its associations with health outcomes at baseline. (A) Taxa that associated with general health at species level (FDR < 0.05). (B) Taxa that associated with general health at genus level (FDR < 0.05). (C) Violin plots for balance score among individuals with sexually transmitted infections, clinical symptoms and vaginal or cervical diseases, comparing with those without any reported above phenotypes. (D) Line chart indicate the changes of balance score (species and genus) with aging. (E) Mediation analysis revealed the role of balance score in the association between lifestyle and general health. The red and gray lines represent forward and reverse mediations respectively. (F) The significant mediation effect, and the proportion of forward mediation is indicated in the middle of the triangle. The color of the edge represents positive (red) and negative (blue) correlation. *** on the edge represent the significance of the correlation between two ends of the edge. P (ADE), *p* value of the average direct effect. (G) Extrapolation of balance score (species) to 13 vaginal microbiome case-control studies. For each dataset, the balance score (species) between disease and control is shown. *p* value was obtained by Wilcoxon rank-sum test. **** *p* < 1e-4, *** *p* < 0.001, ** *p* < 0.01, * *p* < 0.05, NS, not significant. Detailed information is provided in Table S14-17.


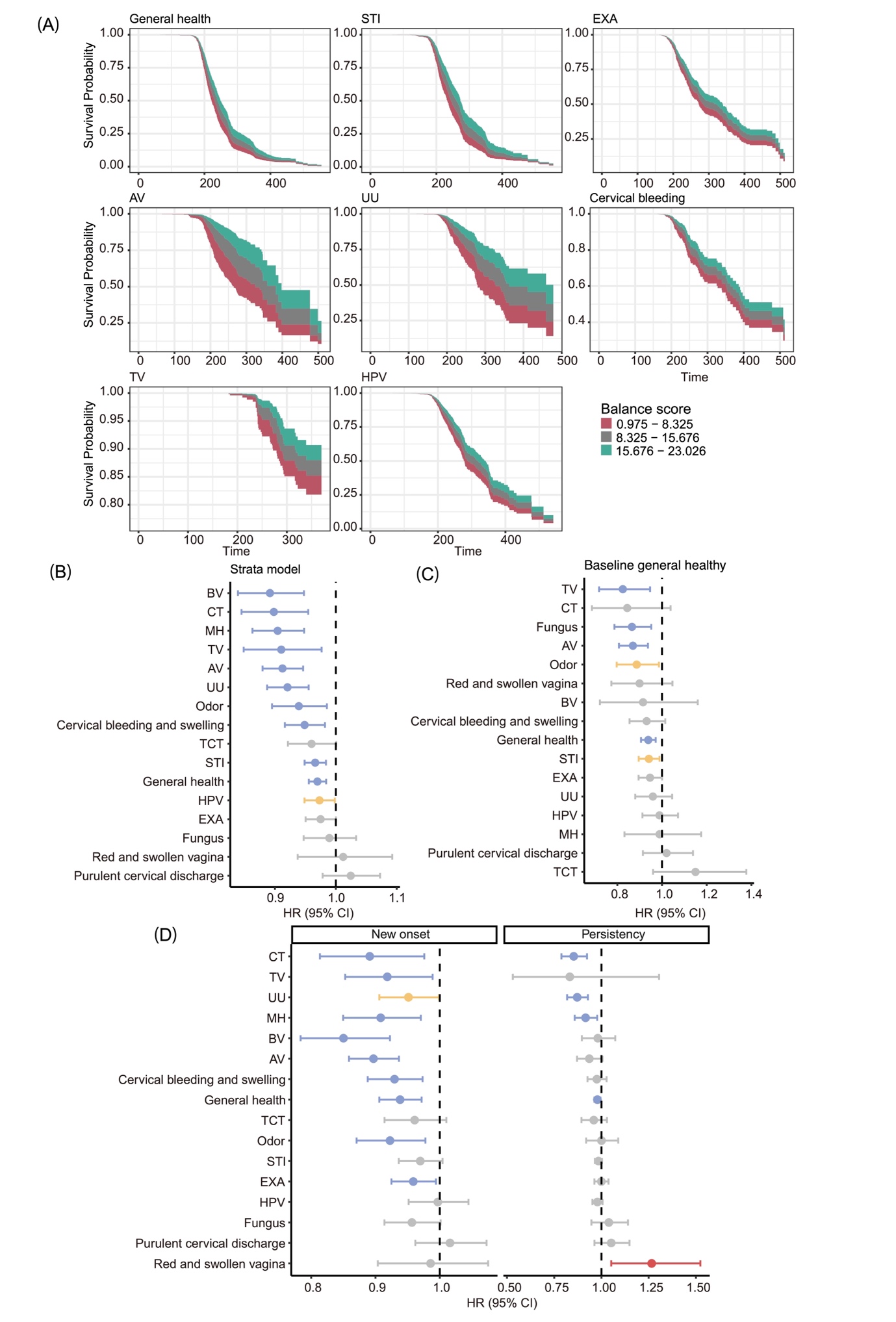


## Figure S8 Vaginal microbiome balance score and its associations with health outcomes at baseline. (A) Survival plots for features with significant associations in Cox regression. The area under the curve represents the probability of the outcome over time, with color gradients indicating values of the continuous balance score. (B) Associations between baseline balance score and follow-up health outcomes adjusting for baseline status of health outcome. (C) Associations between baseline balance score and follow-up health outcomes in subset of women who were general healthy at baseline. (D) Subgroup analyses in participants stratified based on baseline disease status, including two subgroups: "new onset" (outcome-negative at baseline) and "persistence" (outcome-positive at baseline). Hazard ratios (HRs) with 95% CIs and *p* values are shown from Cox regression models. Significant associations (FDR < 0.05) are highlighted in blue, nominal significant results (*p* < 0.05) are highlighted in yellow; non-significant results are shown in gray. Detailed information is provided in Table S19.


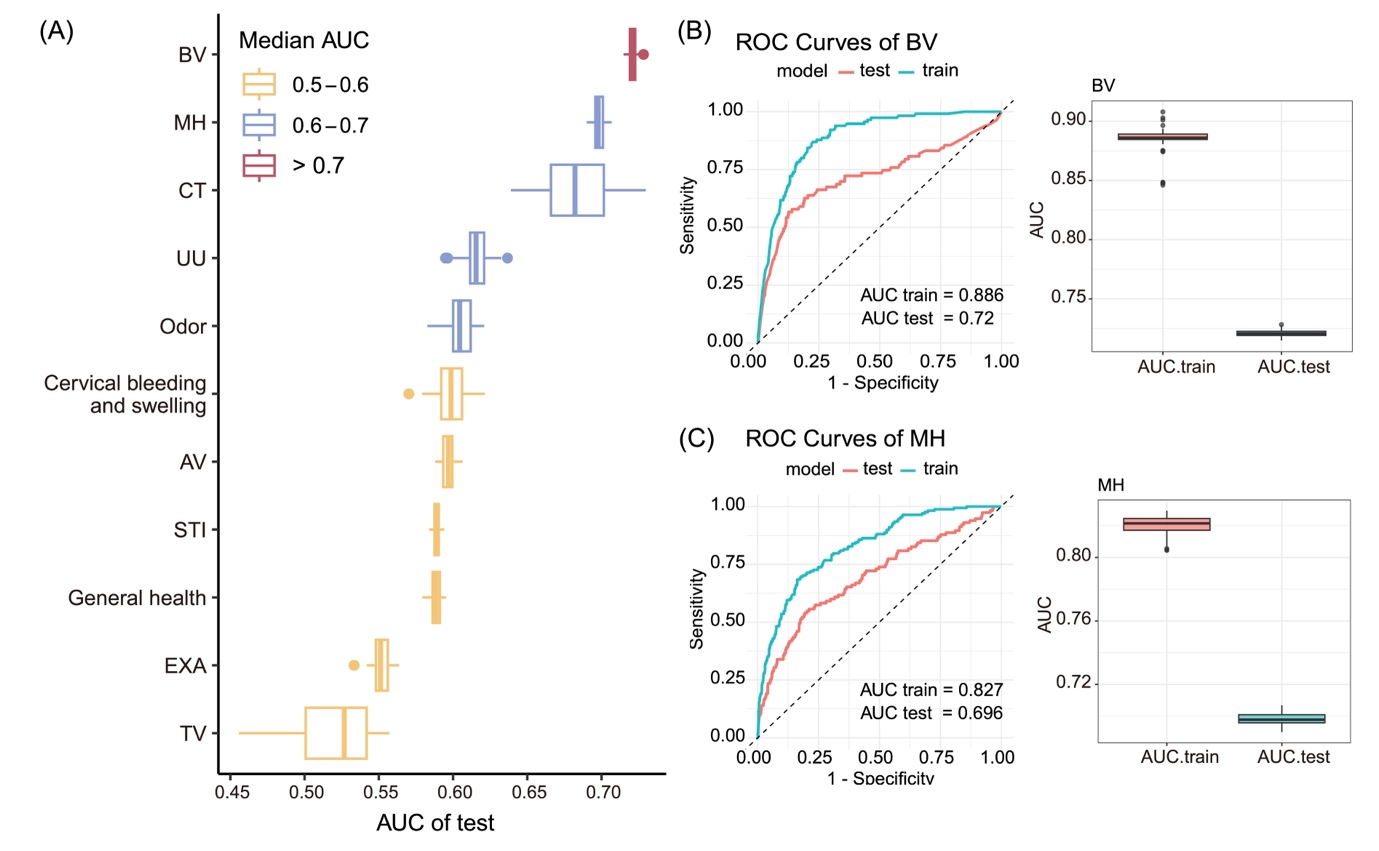


## Figure S9 Baseline microbiome balance score predicts future vaginal health outcomes. (A) Boxplot showing the area under the curve (AUC) of prediction models for future health outcomes based on baseline balance score. Models were trained on baseline data and tested on follow-up data. (B-C). Receiver operating characteristic (ROC) curves and AUC distributions for models predicting bacterial vaginosis (BV) and *Mycoplasma hominis* (MH). The blue line represents model performance in the training set; the red line represents the test set.


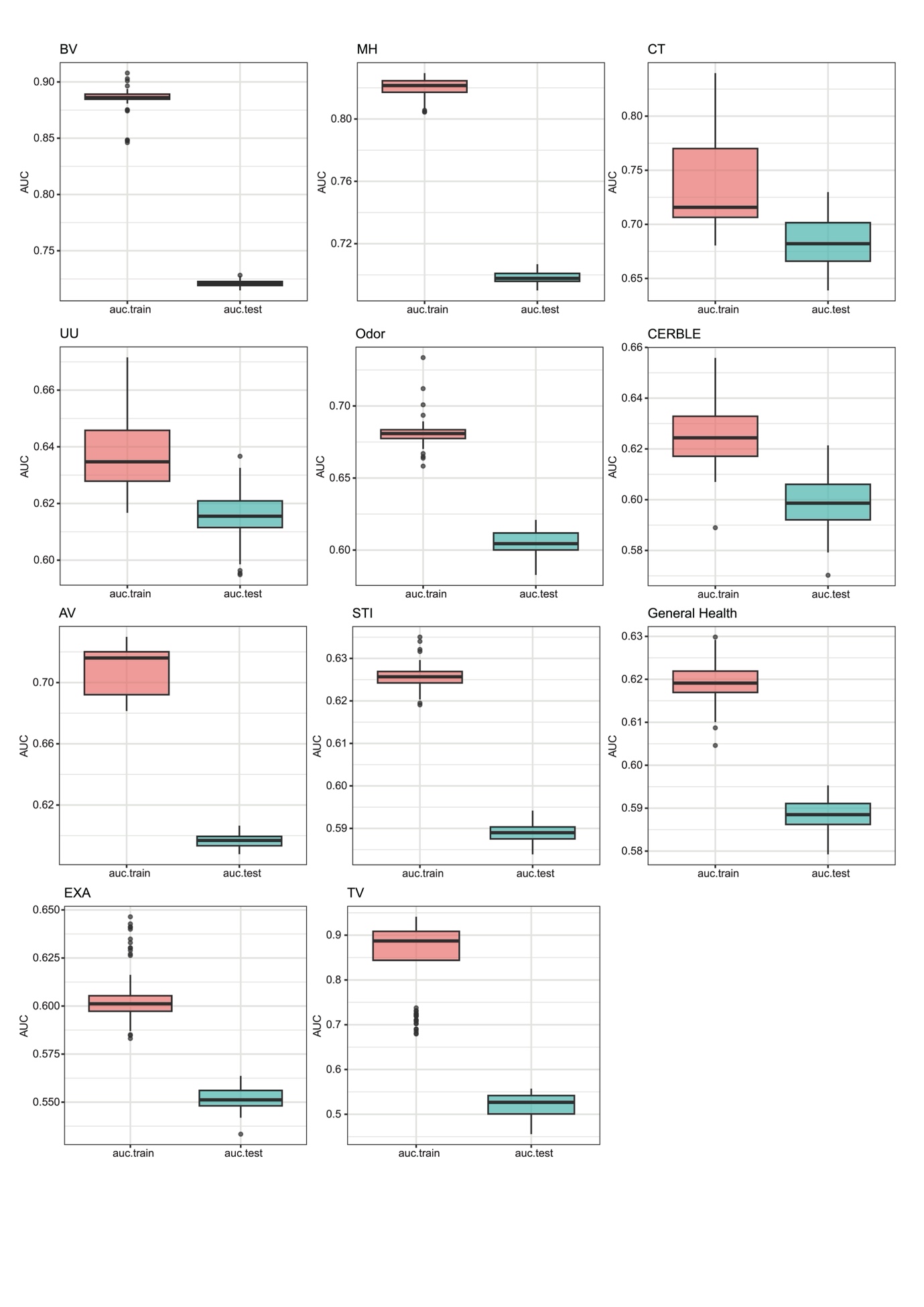


## Figure S10 AUC distributions for models predicting health outcomes at follow-up. Boxplot showing the area under the curve (AUC) of prediction models for future health outcomes based on baseline balance score. Models were trained on baseline data and tested on follow-up data. BV = Bacterial vaginosis. MH = Mycoplasma hominis. CT = Chlamydia trachomatis. UU = Ureaplasma urealyticum. CERBLE = Cervical bleeding and swelling. AV = Aerobic vaginitis. STI = Sexually transmitted infection. EXA = Symptoms recorded by clinical examination. TV = Trichomonas vaginalis.

## CALM2004 Consortium Investigators

Bingbing Xiao (Peking University First Hospital, Beijing, China)

Shuyi Han (Jinan central Hospital Affiliated to Shandong First Medical University, Jinan, China)

Fen Lin, Yonghao Wu (Chaozhou Central Hospital, Chaozhou, China)

Changzhong Li (Shandong Provincial Hospital Affiliated to Shandong First Medical University, Jinan, China)

Ruizhe Wang (The First Hospital of China Medical University, Shenyang, China)

Weiguang Luo, Jing Zhao (Henan Provincial people’s Hospital, Zhengzhou, China)

Xiaoyan Li (The Fifth Affiliated Hospital of Southern Medical University, Guangzhou, China)

Liangzhi Cai, Pengming Sun (Fujian Maternity and Child Health Hospital, Fuzhou, China)

Fan Lu (The Affiliated Hospital of Guizhou Medical University, Guiyang, China)

Wenjun Zhu, Yingxiu Chen (The Second People's Hospital of Lianyungang, Lianyungang, China)

Bin Huang (The First Affiliated Hospital of Sun Yat-sen University, Guangzhou, China)

Zhiyu Pang (The First Affiliated Hospital of Guangzhou University of Chinese Medicine, Guangzhou, China)

Kaifeng Wu (The First People’s Hospital of Zunyi, Zunyi, China)

Yan Han (Changzhi People's Hospital of Changzhi Medical University, Changzhi, China)

Guanghui Chen, Hui Chen (Affiliated Xiaolan Hospital, Southern Medical University, Zhongshan, China)

Beibei Sun (Zhuhai Maternal and Child Health Care Hospital (Zhuhai Women and Children’s Hospital), Zhuhai, China)

Jinbo Liu, Ping Zhan (The Affiliated Hospital of Southwest Medical University, Luzhou, China)

Bo Wang (Hainan General Hospital, Haikou, China)

Haitao Yu (The First Hospital of Lanzhou University, Lanzhou, China)

Qunxiang Liu (Taihe Branch of the Nanfang Hospital, Southern Medical University, Chaozhou, China)

Xuefeng Wang (The Third Affiliated Hospital of Southern Medical University, Guangzhou, China)

Xuesu He (Dazhou integrated TCM&Wester Medicine Hospital, Dazhou, China)

Hong Chen, Shuhua Li (The First Hospital of Qiqihar, Qiqihar, China)

Kewei Zhao (The Third Affiliated Hospital of Guangzhou University of Chinese Medicine, Guangzhou, China)

Xiaojuan Gao (Shenzhen Luohu Hospital Group Luohu People’s Hospital, Shenzhen, China)

Yanan Zhang (Hongqi Hospital Affiliated to Mudanjiang Medical University, Mudanjiang, China)

Xianjin Wu, Maocheng Li (Huizhou Central People's Hospital, Huizhou, China)

Guijie Zhang (Jilin People’s Hospital, Jilin, China)

Meiling Luo (The First Affiliated Hospital of Guangxi Medical University, Nanning, China)

Liang Peng (The Fifth Affiliated Hospital of Guangzhou Medical University, Guangzhou, China)

Ming Li (The first affiliated hospital of university of USTC, Hefei, China)

Zhijuan Liu (Tibet Autonomous Region People's Hospital, Lasa, China)

Yufeng Xiong (Nanfang Hospital, Southern Medical University, Guangzhou, China)

Jing Sha (Maternal and Child Health Care Hospital of Inner Mongolia Autonomous Region, Huhehot, China)

Chaoxin Jiang (Guangdong Hospital of Traditional Chinese and Western Medicine, Foshan, China)

Xue Guo (The Seventh Affiliated Hospital, Sun Yat-sen University, Shenzhen, China)

Lianhua Wei (Gansu Provincial Hospital, Lanzhou, China)
